# Strength in Cognitive Self-Regulation

**DOI:** 10.3389/fpsyg.2013.00174

**Published:** 2013-04-11

**Authors:** Ayla Barutchu, Olivia Carter, Robert Hester, Neil Levy

**Affiliations:** ^1^Florey Institute of Neuroscience and Mental Health, The University of MelbourneParkville, VIC, Australia; ^2^Psychological Sciences, The University of MelbourneParkville, VIC, Australia; ^3^Oxford Centre for Neuroethics, The University of OxfordOxford, UK

**Keywords:** self-control, depletion, response inhibition, arithmetic task, self-regulation, task switching

## Abstract

Failures in self-regulation are predictive of adverse cognitive, academic and vocational outcomes, yet the interplay between cognition and self-regulation failure remains elusive. Two experiments tested the hypothesis that lapses in self-regulation, as predicted by the *strength model*, can be induced in individuals using cognitive paradigms and whether such failures are related to cognitive performance. In Experiments 1, the stop-signal task (SST) was used to show reduced behavioral inhibition after performance of a cognitively demanding arithmetic task, but only in people with low arithmetic accuracy, when compared with SST performance following a simple discrimination task. Surprisingly, and inconsistently with existing models, subjects rapidly recovered without rest or glucose. In Experiment 2, depletions of both go-signal reaction times and response inhibition were observed when a simple detection task was used as a control. These experiments provide new evidence that cognitive self-regulation processes are influenced by cognitive performance, and subject to improvement and recovery without rest.

## Introduction

Self-regulation is fundamental to human function, with many psycho-social problems, including drug addiction, obesity, and gambling, being directly linked to lapses in the capacity to maintain control over behavior and function. It is widely believed to be dependent on a range of dissociable processes, including attention, decision-making, volition, and the inhibition of unwanted impulses. Recently, there has been a surge of research in support of the *strength model of self-regulation* (for meta-analysis see Hagger et al., 2010). According to this model, self-regulation across various domains, ranging from cognition to social processes, draws upon a common resource that depletes with use (Baumeister et al., 1998; Baumeister and Vohs, 2007), and replenishes with rest or glucose intake (Gailliot and Baumeister, 2007; Gailliot et al., 2007; Tyler and Burns, 2008). On a wide range of tasks requiring self-regulation, performance suffers if the task is preceded by another also requiring self-regulation even if the tasks are unrelated. For instance, participants will persist with squeezing a handgrip for a significantly shorter period of time after having to control their emotions while watching a sad movie (Muraven et al., 1998). The depletion of control resource has been reported in other species too (Miller et al., 2010). Many researchers believe that resource depletion partially explains many cases of lapses in self-regulation, ranging from ordinary overeating through to addictive behaviors, and impulsive violence (e.g., Vohs and Heatherton, 2000; Baumeister et al., 2006; Baumeister and Tierney, 2011; Hofmann et al., 2012).

The ability to self-regulate has been associated with better performance on cognitive tasks and also with vocational and academic success (Mischel et al., 1989; Goldberg and Grandey, 2007; Jonker et al., 2010). Prior engagement in self-regulation (e.g., emotional control) can impede later cognitive performance (Schmeichel et al., 2003; Schmeichel, 2007). Higher fluid intelligence has been associated with greater depletion (Shamosh and Gray, 2007). However, of particular concern are the existing inconsistencies in the way cognitive paradigms are employed in self-regulation studies and the outcomes they yield. For example, in dual task paradigm experiments, arithmetic tasks have been used both as controls for depletion tasks that require relatively greater levels of self-regulation (Muraven et al., 1998, 2006), and as follow-up tasks to assess the effects of resource depletion on self-regulation (Wright et al., 2003, 2008; Vohs et al., 2005). There is independent evidence that arithmetic tasks, particularly those that involve continual switching between different problem types (e.g., additions and subtractions), are associated with the depletion of cognitive resources such as executive control and working memory (Schneider and Anderson, 2010). In contrast, researchers studying resource depletion have argued that simple arithmetic problems are over learnt and automated in adults, and that the level of self-regulation engaged by such tasks can be expected to be dependent on individual ability (Baumeister and Tierney, 2011). However, the hypothesis that the depleting effects of such cognitive tasks are dependent on individual performance levels has not been tested.

There are a number of factors that have been identified as having a replenishing effect on self-regulation. Glucose intake (e.g., Gailliot et al., 2007), rest, and sleep are often associated with better self-regulation ability. Even brief periods of rest, less than 10 min, can result in the replenishment of self-regulation resources (Baumeister et al., 2006; Tice et al., 2007; Tyler and Burns, 2008). Given that people often have to maintain high levels of cognitive regulation for extended periods in daily life (e.g., undergraduate students often have to sit though 1 h lectures and maintain a high level of cognitive regulation), questions about the dynamics of self-regulation throughout continual task performance are important. Self-regulation has generally been explored using very brief single episode tasks (i.e., less than 10 min in duration). No study has used repeated measures across several blocks of the same task to assess the stability of cognitive self-regulation resources over time.

To date most studies of resource depletion have also relied on between group designs and measures of persistence to demonstrate that self-regulation is depletable. Such persistence measures are limited in their ability to inform whether worsened performance is caused by a decrease in subjects’ motivation or by an impaired capacity to self-regulate (Levy, 2011). Indeed people are less likely to show depletion if motivated or given incentives to maintain self-regulation (Muraven and Slessareva, 2003; Stewart et al., 2009), or if people believe, or are led to believe, that self-regulation capacity is unlimited (Job et al., 2010). An aim of this study was to provide data about how self-regulation is engaged within the cognitive domain in individuals when discontinuing the task is no longer perceived as a salient option by subjects. We also aimed to investigate whether the depletion of self-regulation within the cognitive domain is influenced by individual differences beyond those highlighted by Job et al. (2010).

In the present study, we took advantage of the natural widespread variability in arithmetic performance, and used a switching arithmetic task that had previously been shown to have depleting effects on cognitive resources (Schneider and Anderson, 2010). It is well accepted within the cognitive literature that both arithmetic and “switching” tasks are highly dependent on cognitive control mechanisms, including the ability to shift attention and regulate information in working memory (e.g., Kiesel et al., 2010; Schneider and Anderson, 2010); it is hypothesized that switching arithmetic tasks can deplete cognitive resources. We classified individuals based on their performance on the arithmetic task, and used the stop-signal task (SST) to investigate whether such cognitive depletion can influence the capacity to regulate and inhibit proponent motor responses to “go” signals following infrequent “stop” signals. Our principal aim was to investigate the relationship between performance and self-regulation. The SST has been widely used to investigate the behavioral and neural processes of cognitive control and response inhibition in various populations (Logan et al., 1997; Band et al., 2003; Alderson et al., 2007). If self-regulation depletion within the cognitive domain is dependent on task performance and cognitive ability, as predicted by Baumeister and colleagues, than the depletion of self-regulation should only be observed in individuals with low performance accuracy. It was also hypothesized that once resources are depleted, self-regulation will not recover across consecutive blocks of the SST without rest.

## Experiment 1

### Materials and methods

#### Participants

Thirty-eight healthy adult volunteers participated in this study (10 males and 28 females, *M* Age = 21; 6 and SD = 3; 5). Age is denoted in years; months. Participants were recruited using poster advertisements. All participants had normal or corrected to normal vision, normal hearing and no prior history of neurological or psychiatric disorders. Participants were paid AUD $ 20 for their time.

#### Self-regulation depletion – arithmetic tasks

**Figure 1:**
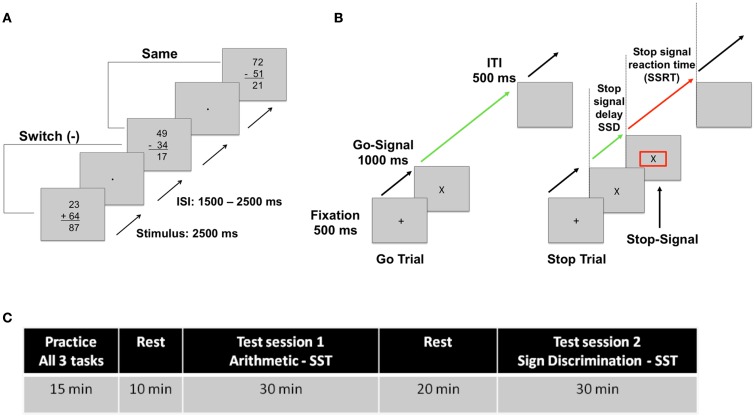
**(A)** A single trial of the arithmetic and simple sign discrimination task. **(B)** A go-trial and a strop-trial of the stop-signal task. **(C)** Experimental protocol timeline.

The effects of resource depletion were investigated using a *switching arithmetic task*, which included two types of simple problems: no-carry addition problems (e.g., 52 + 23 = 75), and no-borrow subtraction problems (e.g., 47–16 = 31). Equations switched randomly between addition and subtraction problems. On half the trials, the answers presented were correct, and on the remaining half incorrect. Incorrect problems were randomly calculated as either ±2 or ±9 relative to the correct answer. All three terms of correct and incorrect problems included double-digit numbers, allowing for the generation of a large sample of unique problems: 992 additions and 1008 subtraction problems fit these criteria (excluding problems with multiples of 10 in the first two terms). Arithmetic problems were presented vertically (see Figure 1A) for a duration of 2.5 s with an inter-stimulus interval (ISI) randomly varying between 1.5 and 2.5 s in four blocks of 48 trials. Correct and incorrect addition and subtraction problems were selected from all possibilities, and presented in random order with an equal probability. The duration of each block was approximately 3 min. Between blocks, participants were given the option of a very short break (<1 min) to stretch or re-adjust if needed. Total duration of the task was about 13 min.

For the resource depletion condition, participants were asked to solve the problems and to indicate whether the answer was correct or incorrect by pressing keys with their index and middle fingers. The control version of the task, which will be referred to as the *sign discrimination task*, was identical to the arithmetic task except that participants were asked to ignore the numbers and the solution to the problems, and indicate the operation type (i.e., whether the operation is an addition or a subtraction). This ensured that stimulus properties and overall task durations were identical for the arithmetic task and the control sign discrimination task.

#### The stop-signal performance task

Following the arithmetic task and the control sign discrimination task, participants’ performance was assessed using the SST (see Figure 1B) (Logan et al., 1997). Participants were asked to respond rapidly to two “go” signals, “X” and “O,” using their index and middle fingers. On 25% of trials, participants were presented with a “stop-signal” consisting of a red box, which surrounded the go-signal. The onset of the “stop-signal” was delayed such that participants were only able to inhibit their responses on approximately 50% of trials. This delay time is referred to as the stop-signal delay (SSD). The initial SSD was set at 250 ms and incremented or decremented by 50 ms thereafter depending on the participant’s success and failure at inhibition, to maintain an overall stopping rate (i.e., a correct response inhibition rate) at approximately 50%. Only participants with an overall accuracy rate above 40% and below 60% on each block of the task were included in further data analyses (two participants failed this standard inclusion criterion). The stop-signal reaction time (SSRT) was calculated as the difference between the go-signal motor RT (go-RT) and the SSD at which participants could inhibit approximately 50% of responses (SSRT = go-RT − SSD). The go-RT and the SSRT were measured across three consecutive blocks of 100 trials, which took approximately 15 min to complete (5 min per block). At the end of each block, participants were provided with feedback on their accuracy, go-RT and SSRT, and the option of a very short break (<1 min) if needed.

#### Procedure

For this experiment a repeated measures paradigm was employed. Participants were seated in a quiet room at a distance of 1 m from a 17″ computer monitor. In the initial practice session, participants were given practice runs to familiarize themselves with all three tasks: the arithmetic task, the sign discrimination task and the SST. For the sign and arithmetic tasks, participants were allowed up to 48 trials of practice. For the SST, all participants were given three practice blocks of 25 trials. Practice sessions lasted approximately 10–15 min and were followed by a 10 min rest period. During the rest period, participants were asked to complete a brief 1 min demographic questionnaire. For the remainder of the time, participants were encouraged to rest and engage in activities they found relaxing. The rest period was followed by the first test session, during which participants were asked to complete either the arithmetic or the sign discrimination task followed by the SST. In the second test session, participants were asked to complete the alternate task followed by the SST. The order of the arithmetic and the sign discrimination tasks was counterbalanced across participants. Each test session lasted approximately 30 min. The two test sessions were separated by a 20 min rest period. During both rest periods, participants were played relaxing classical music (solo piano pieces composed by Eric Satie) to minimize any depleting effects of the preceding tasks (Tyler and Burns, 2008). See Figure 1C for experimental protocol timeline.

### Results

Performance across the four blocks of the arithmetic task was highly correlated for both RTs (*r* > 0.85, *p* < 0.001 for all blocks) and accuracy (*r* > 0.6, *p* < 0.001 for all blocks) measures. To investigate whether differences in cognitive performance influenced the depletion of self-regulation resources, participants were subdivided into two groups based on their performance on the arithmetic task: a high accuracy group including participants whose accuracy measures approached ceiling, with a total error rate below 5% (*n* = 20, *M* error rate = 3.14, SEM = 0.29), and a low accuracy group with total error rates above 5% (*n* = 15, *M* error rate = 10.54, SEM = 1.24). The high accuracy group consisted of 10 males and 10 females (*M* age = 21; 7, SD = 3; 5), and the low accuracy group consisted of 15 females (*M* age = 21; 2, SD = 2; 5). Error rates for the high and low accuracy groups on the sign discrimination task floored (averaging < 4% with the exception of one participant with an overall error rate of 24% who was excluded from all further data analyses).

The pattern of choice RTs differed between the sign and arithmetic tasks. Overall, RTs for the detection of additions (low accuracy group *M* = 597, SEM = 24.66, and high accuracy group *M* = 573, SEM = 21.76) and subtractions (low accuracy group *M* = 586, SEM = 20.36, and high accuracy group *M* = 579, SEM = 21.72) did not significantly differ on the sign task. In contrast, for the arithmetic task a two-way Analysis of Variance (ANOVA) showed RTs to be significantly faster for additions (low accuracy group *M* = 1445, SEM = 89.52, and high accuracy group *M* = 1372, SEM = 65.81) than subtractions (low accuracy group *M* = 1615, SEM = 100.18, and high accuracy group *M* = 1505, SEM = 68.14), *F*(1,33) = 56.28, *p* < 0.001, η*^2^* = 0.63. Although RTs were faster for the high than the low accuracy group, this difference did not reach significance.

**Figure 2:**
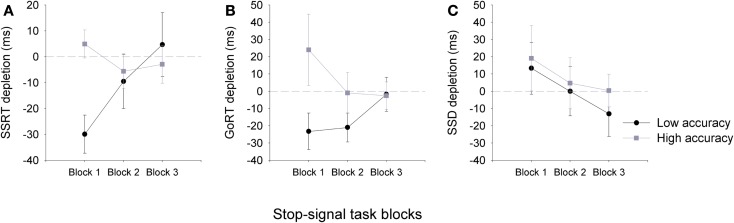
**Mean (±SEM) depletion measures for SSRT (A), go-RT (B), and SSD for the low accuracy (black line) and high accuracy (gray line) (C) groups across the three blocks of the stop-signal task: Block 1 (0–5 min), Block 2 (5–10 min), and Block 3 (10–15 min)**. Depletion measures were calculated by subtracting SST task measures following the arithmetic task from those that followed the sign task (e.g., SSRT depletion = SSRT sign – SSRT arithmetic), therefore, negative values are representative of a depletion effect for SSRT and go-RT measures following the arithmetic task (note that for SSD a negative value is representative of an improvement in performance). Dashed gray line at zero depicts no depletion or gain in performance.

To examine resource depletion in our low and high accuracy groups, a depletion measure was calculated for each block of the SST by subtracting task performance measures following the arithmetic task from those that followed the sign task (i.e., SSRT depletion = SSRT following sign task – SSRT following arithmetic task). As can be observed in Figure 2, the arithmetic task did not have a depleting effect on individuals with high accuracy. In contrast, for individuals in the low accuracy group, the arithmetic task did have a depleting effect on SSRT measures, but only on the first block of the SST. A three-way 2(group) × 2(task) × 3(block) ANOVA showed a significant three-way interaction, *F*(2,66) = 4.13, *p* = 0.02, η*^2^* = 0.11. Follow-up *post hoc* analyses showed that SSRTs for block 1 were significantly slower for the low accuracy group following the arithmetic task, but not the high accuracy group. A significant correlation was also observed for SSRT depletion and overall arithmetic task accuracy at block 1 (*n* = 35, *r* = −0.36, *p* < 0.05), but not block 2 (*n* = 35, *r* = −0.21, *p* > 0.05) or block 3 (*n* = 35, *r* = −0.02, *p* > 0.05).

Go-signal RTs and SSD measures were also affected by the preceding task. A three-way ANOVA for go-RT, *F*(2,66) = 4.56, *p* = 0.01, η*^2^* = 0.12, and SSD, *F*(2,66) = 4.31, *p* = 0.02, η*^2^* = 0.12, measures showed a significant two-way interaction between group and SST block. Go-RTs and SSD measures did not differ between groups, however, for the low accuracy group the SSD significantly increased at block 2, and at both blocks 2 and 3 for go-RT (see Table 1). The ANOVA main effects and the three-way interactions for the SSD and go-RT did not approach significance. For the SST task accuracy measures did not significantly differ between the groups (see Table 1).

**Table 1:** **Mean (±SEM) percentage (%) accuracy (ACC), motor reaction times for go-signals (go-RT), stop-signal delays (SSD), and stop-signal reaction times (SSRT) for the three blocks of the stop-signal task following the sign discrimination task and the arithmetic task in Experiment 1**.

	Sign discrimination task				Arithmetic task			
	ACC	Go-RT	SSD	SSRT	ACC	Go-RT	SSD	SSRT
**LOW**								
Block 1	49.87 ± 1.23	453 ± 17.10	230 ± 21.20	223 ± 10.24	49.33 ± 1.01	470 ± 15.16	217 ± 16.97	253 ± 11.01
Block 2	50.04 ± 0.53	480 ± 17.89	248 ± 19.91	232 ± 9.45	49.73 ± 0.67	490 ± 18.85	248 ± 20.75	242 ± 12.93
Block 3	49.69 ± 0.63	486 ± 18.78	243 ± 18.16	242 ± 9.45	50.22 ± 0.41	494 ± 19.76	256 ± 24.34	237 ± 13.53
**HIGH**								
Block 1	49.50 ± 1.01	475 ± 29.93	240 ± 23.18	235 ± 9.49	48.80 ± 0,62	452 ± 11.54	222 ± 12.68	230 ± 6.25
Block 2	49.53 ± 0.60	454 ± 12.86	224 ± 20.32	230 ± 11.44	49.80 ± 0.46	454 ± 9.94	219 ± 14.50	235 ± 10.07
Block 3	49.78 ± 0.30	458 ± 10.49	217 ± 17.08	241 ± 11.69	49.75 ± 0.26	461 ± 11.72	217 ± 17.20	244 ± 12.96

### Discussion

The outcomes of Experiment 1 suggest that the depletion of self-regulation resources is partly dependent on one’s cognitive performance. This finding is consistent with the suggestion that well learned tasks are likely to become automated, thereby engaging few self-regulation resources (Baumeister and Tierney, 2011). Interestingly, contrary to the predictions of the strength model, impairment of SSRT appeared to recover by the second block of the SST, however, this recovery was at the expense of go-signal motor speed; motor RTs were significantly slower for blocks 2 and 3 for the low accuracy group, but not the high accuracy group.

By nature, cognitive tasks, even those as simple as our sign discrimination task, engage multiple capacities involved in self-regulation (e.g., sustained attention, memory, executive control, etc), which could act as potential confounds by affecting self-regulation resources. Furthermore, in Experiment 1 participants were presented with solvable equations in the control sign discrimination task. Although participants were asked to ignore the solutions of problems and only report the sign of the equation, it is possible that participants were inadvertently solving these tasks as they had adequate time due to the stimulus presentation times being held constant across the depletion and control tasks.

Experiment 2 aimed to replicate the findings of Experiment 1 using a simple detection task with nonsensical equations as the control non-depleting task in place of the sign discrimination task, to remove the possibility that the observed effects are influenced by subjects automatically solving the equations or processing the symbolic meaning of the “sign” in the control task. It was hypothesized that self-regulation depletion will be only observed for participants with low performance accuracy on the arithmetic task. Based on the outcomes of Experiment 1, it was also posited that self-regulation will recover across consecutive blocks of the SST without rest.

## Experiment 2

### Materials and methods

#### Participants and procedure

Forty-eight adults participants (14 males and 34 females, *M* Age = 23, 11 and SD = 6; 5 years; months) were subdivided into two groups based on their performance on the arithmetic task: low accuracy groups with error rates > 5% (*n* = 30, *M* error rate = 3.05, SEM = 0.36) and a high accuracy group with error rates < 5% (*n* = 18, *M* error rate = 11.61, SEM = 1.10). The low accuracy group included 23 females and 7 males (*M* age = 23; 11, SD = 6; 5), while the high accuracy group consisted of 11 females and 7 males (*M* age = 22; 1, SD = 3; 7) Participants were paid AUD$20 for their time.

All procedures for Experiment 2 were as in Experiment 1 with the exception of the sign discrimination task, which was replaced with a simple detection task. For the simple detection task, the same equations were presented as in the arithmetic task, however, all numbers were randomly replaced with (unreadable) capital consonant letters to ensure that problems were unsolvable. The order of the detection task and the arithmetic task was counterbalanced across participants. Participants were instructed to randomly press buttons with their middle and index finger upon the detection of an equation.

In Experiment 2, some participants reported feeling fatigued following the simple detection task, therefore we introduced subjective measures completed by the last 42 participants. To assess level of alertness, we used the Stanford Sleepiness Scale (SSS) (Glenville and Broughton, 1978). Visual analog scales (VAS), 10 cm in length, were also used to obtain participant ratings of (1) how relaxed they felt (very relaxed – very stressed), (2) how alert they felt (very tired/sleepy – very alert), (3) how easy it was to concentrate (very easy to concentrate – very difficult to concentrate), and (4) how difficult the task was (very easy – very difficult). Subjective measures took less than 1 min to complete at the end the arithmetic and simple detection task (i.e., prior to commencing the SST).

### Results

Performance across the 4 blocks of the arithmetic task was highly correlated for both RTs (*r* > 0.8, *p* < 0.001 for all blocks) and accuracy (*r* > 0.58, *p* < 0.001 for all blocks) measures. For the simple detection task, error rates floored, averaging below 4%, and motor RTs did not significantly differ between the high (*M* = 401, SEM = 17.27) and low (*M* = 448, SEM = 25.53) accuracy groups, *t*(46) = −1.11, *p* > 0.05. For the arithmetic task, using a two-way ANOVA RTs were found to be faster for additions (low accuracy group *M* = 1489, SEM = 53.57, and high accuracy group *M* = 1265, SEM = 54.35) than subtractions (low accuracy group *M* = 1637, SEM = 65.42, and high accuracy group *M* = 1390, SEM = 60.81), *F*(1,46) = 83.64, *p* < 0.001, and participants in the high accuracy groups were significantly faster at solving question than those in the low accuracy groups, *F*(1,46) = 7.14, *p* < 0.01.

**Figure 3:**
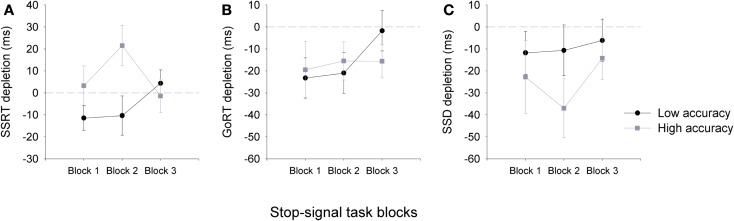
**Mean (±SEM) depletion measures for SSRT (A), go-RT (B) and SSD (C), for the low accuracy (black line) and high accuracy (gray line) groups across the three blocks of the stop-signal task: Block 1 (0–5 min), Block 2 (5–10 min), and Block 3 (10–15 min)**. Depletion measures were calculated by subtracting SST task measures following the arithmetic task from those that followed the sign task (e.g., SSRT depletion = SSRT sign – SSRT arithmetic), therefore, negative values are representative of a depletion effect for SSRT and go-RT measures following the arithmetic task (note that for SSD a negative value is representative of an improvement in performance). Dashed gray line at zero depicts no depletion or gain in performance.

A significant three-way interaction was observed for SSRT impairment using a 2(group) × 2(task) × 3(block) ANOVA, *F*(2,92) = 3.15, *p* < 0.05. The depletion of SSRT measures was only observed for the low accuracy groups, which recovered by the third block of the SST (Figure 3). Interestingly, a significant difference between the accuracy groups was observed for block two only, where participants in the high accuracy groups seem to show a gain in their SSRT following the arithmetic task. Consistent with Experiment 1, significant correlations were also observed between overall measures of accuracy on the arithmetic task and measures of SSRT depletion at block 1 (*n* = 48, *r* = −0.26, *p* < 0.05) and block 2 (*n* = 48, *r* = −0.39, *p* < 0.01), but not block 3 (*n* = 48, *r* = 0.04, *p* > 0.05).

Reaction times for go-signals were also affected by the preceding task (Table 2). A three-way ANOVA for go-RTs of SST showed a significant main effect of task and group. RTs were significantly slower for go-signals following the arithmetic task than the simple detection task, *F*(1,92) = 9.99, *p* = 0.003, and overall RTs for the low accuracy group were slower than the high accuracy group, *F*(1,46) = 6.41, *p* = 0.02. A three-way ANOVA revealed a significant main effect of task for SSD measures too. SSDs were significantly increased following the arithmetic task, *F*(1,92) = 6.36, *p* = 0.02, however, SSD did not significantly differ between the groups.

**Table 2:** **Mean (± SEM) percentage (%) accuracy (ACC), motor reaction times for go-signals (go-RT), stop-signal delays (SSD), and stop-signal reaction times (SSRT) for the three blocks of the stop-signal task following the simple detection task and the arithmetic task in Experiment 2**.

	Simple detection task				Arithmetic task			
	ACC	Go-RT	SSD	SSRT	ACC	Go-RT	SSD	SSRT
**LOW**								
Block 1	50.93 ± 0.63	484 ± 13.61	247 ± 13.66	237 ± 5.31	50.40 ± 0.65	507 ± 13.07	259 ± 13.01	248 ± 4.15
Block 2	50.13 ± 0.30	481 ± 11.32	243 ± 13.65	238 ± 5.19	50.40 ± 0.41	502 ± 13.07	254 ± 11.55	248 ± 7.41
Block 3	50.13 ± 0.22	496 ± 12.23	248 ± 12.77	248 ± 5.33	50.13 ± 0.19	498 ± 9.63	254 ± 11.54	244 ± 6.47
**HIGH**								
Block 1	48.00 ± 0.86	431 ± 12.62	207 ± 16.71	224 ± 8.13	49.33 ± 0.92	450 ± 15.08	230 ± 19.11	220 ± 8.67
Block 2	49.33 ± 0.49	443 ± 16.38	199 ± 21.18	244 ± 9.30	49.22 ± 0.46	459 ± 18.81	236 ± 22.71	223 ± 10.78
Block 3	49.70 ± 0.32	450 ± 15.41	216 ± 22.41	233 ± 10.12	49.11 ± 0.39	465 ± 16.88	231 ± 21.51	235 ± 9.79

An assessment of subjective measures, using a series of two-way ANOVAs, showed that participants scored significantly higher on the SSS following the simple detection task, *F*(1,39) = 16.22, *p* < 0.001 (see Table 3). VAS also indicated that participants were significantly more relaxed, *F*(1,40) = 11.44, *p* = 0.002, and less alert, *F*(1,40) = 9.58, *p* = 0.004 following the simple detection task than the arithmetic task. Subjective measures of concentration did not differ between tasks and groups. However, accuracy group and proceeding task significantly interacted for the subjective measure of difficulty, *F*(1,40) = 6.53, *p* = 0.01. Interestingly, participants in the low accuracy group rated the simple detection task to be significantly easier than the high accuracy group. For the high accuracy group, subjective difficulty measures did not significantly differ between the arithmetic and simple detection task.

**Table 3:** **Mean (± SEM) subjective measures for the Stanford Sleepiness Scale (SSS), and measure on the visual analog scales (VAS) for relaxation, alertness, concentration and task difficulty**.

	Simple Detection		Arithmetic	
	Low Accuracy	High Accuracy	Low Accuracy	High Accuracy
SST	3.52 ± 0.27	3.57 ± 0.33	2.37 ± 0.22	2.71 ± 0.34^∧^
VAS – relaxation	1.61 ± 0.26	2.58 ± 0.30	3.31 ± 0.48	3.60 ± 0.45^∧^
VAS – alertness	4.16 ± 0.46	3.89 ± 0.58	6.28 ± 0.51	4.96 ± 0.49^∧^
VAS – concentration	4.43 ± 0.57	3.89 ± 0.39	3.30 ± 0.42	3.79 ± 0.53
VAS – difficulty	0.80 ± 0.24	2.24 ± 0.54	3.46 ± 0.46	2.63 ± 0.49*

*Note*. ^∧^for a significant main effect of task, * for a significant interaction effect between task and accuracy group.

*For VAS 10 cm scales were used. Each end of the scale was labeled as follows*.

*Relaxation: very relaxed – very stressed*.

*Alertness: very tired/sleepy – very alert*.

*Concentration: very easy to concentrate – very difficult to concentrate*.

*Difficulty: very easy – very difficult*.

### Discussion

Consistent with the findings of Experiment 1, we observed a significant relationship between SSRT depletion measures on the SST and performance accuracy on the arithmetic task; however in Experiment 2 significant recovery of SSRT depletion was observed by block three of the task. Following the arithmetic task, we also observed an unexpected gain in SSRTs at block 2 for the high accuracy group, but a reverse decrement in response inhibition for the low accuracy group further suggesting that the two accuracy groups are affected differently by the preceding task. RTs to go-signals of the SST were also affected, significantly slowing down following the arithmetic task, suggesting that depletion effects can generalize to other processes.

## General Discussion

The present study used stringent psychophysical measures to demonstrate changes in self-regulation across unrelated cognitive tasks independent of self-driven task persistence. Our results indicate that cognitive tasks can have a temporary depleting effect on unrelated self-regulation processes within individuals; however, such effects are dependent on task performance with some individuals showing a reversal of the effect (i.e., a gain in performance). These changes in self-regulation can generalize to processes other than response inhibition when a simple detection task is used as a control, suggesting that the effects are not limited to task difficulty, and that relatively easy discrimination tasks with minimal cognitive engagement can also influence self-regulation processes.

The extent to which self-regulation is engaged within the cognitive domain is dependent on task performance for individuals. In both experiments, a significant relationship was observed between arithmetic task performance and response inhibition measures. Importantly, evidence of depletion was found within individuals using the SST, which is a robust response inhibition task where accuracy is controlled to the same baseline level and failure to persist is not a salient option, suggesting that it is not just one’s willingness to persist that is affected, but rather that initial effort to self-regulate effects later capacity to self-regulate. This fact makes a motivational explanation of results unlikely. Moreover, the rapidity of recovery, in the absence of any factor that would be expected to affect motivation, seems to indicate that motivation does not explain performance on the SST. In Experiment 1, the observed recovery in SSRT at block 2 and 3 was at the expense of go-signal motor speed. However, go-signal RTs were significantly faster following the simple detection task in Experiment 2, therefore changes in response inhibition (i.e., SSRTs) following the arithmetic relative to the sign discrimination and the simple detection task cannot be attributed to participants’ slowing down to improve response inhibition performance in Experiment 2. In addition, the findings cannot be explained by changes in subjects’ state of alertness since both groups of participants reported greater levels of alertness and less fatigue following the arithmetic task, yet only the low accuracy groups showed poorer regulation of response inhibition in Experiment 2. Also both groups reported similar levels of concentration for both the arithmetic and control task, with only measures of perceived task difficulty significantly differing between the two groups. It has been argued that the level of self-regulation engaged by such tasks is dependent on cognitive performance and ability (Muraven et al., 1998; Baumeister and Tierney, 2011). Consistent with this premise, in the present study only individuals with low accuracy on the arithmetic task showed sign of self-regulation depletion on an unrelated response inhibition task. In previous work, higher fluid intelligence has been associated with greater depletion (Shamosh and Gray, 2007). However, those researchers used a non-cognitive depletion task (an emotion regulation task); participants with high fluid intelligence did not have an advantage over those with lower fluid intelligence with regard to that depletion task. The participants in our experiments with better arithmetical capacity may not have needed to depend to any significant extent on self-regulation processes while performing these tasks. In contrast, those who find arithmetic problems challenging are more likely to find the task effortful and draw upon processes engaging self-regulation, such as executive control and working memory, leading to the overall depletion of available resources.

In the present study, reduced performance on the arithmetic task may reflect differences in arithmetic or task switching “ability,” or a reduced capacity to maintain control or attend during the arithmetic task, resulting in the need for an individual to up-regulate cognitive self-regulation processes. The question of cognitive ability and its interplay with task switching effects is interesting and could be further investigated by using independent measures of performance on a cognitive task. One possible explanation of the present findings is that the observed “depletion” and recovery may be reflective of residual task switch costs, which have been shown to have long lasting effects on performance beyond the immediate switch, with the magnitude of the cost decreasing with practice (e.g., Wylie and Allport, 2000; Monsell, 2003; Berryhill and Hughes, 2009). Individuals who perform poorly on a given task (i.e., in the present case the arithmetic task) may be more prone to such residual task switch costs. Alternatively, the results might also be explained by phasic shifts in attention or some other construct. If that hypothesis were correct, the placement of subjects into groups based on their performance on the arithmetic task and their subsequent SSRT performance might simply reflect such transitory shifts, rather than being the effect of the manipulation. The subsequent improvement in performance could therefore be the product of a recovery from this phasic shift and not the washing-out of depletion. The current results may also be influenced by regression to the mean. This explanation is unlikely, because regression to the mean predicts that participants who score lower at one time are likely to score higher at another and vice versa; therefore, it predicts an improvement in performance following the arithmetic task relative to the control task in the high accuracy group, and vice versa for the low accuracy group. These are possibilities important to pursue in subsequent work, as it is conceivable that they can contribute to the observed results.

At odds with the predictions of the strength model, and with our second hypothesis, self-regulation depletion in the low accuracy group appeared to recover by block 2 of the SST in Experiment 1, and block 3 in Experiment 3, suggesting that response inhibition processes were only temporarily altered with participants quickly regaining and up-regulating their ability to inhibit proponent responses even in the absence of a rest period. However, it is important to note that this recovery was at the expense of response speed to go-signals. More interestingly, in Experiment 2 participants in the high accuracy group, but the not the low accuracy group, showed a significant gain in SSRTs by the second block of the SST. These findings conflict with previous suggestions that motivational incentives (Muraven and Slessareva, 2003; Stewart et al., 2009) or glucose (e.g., Gailliot and Baumeister, 2007; Gailliot et al., 2007) are required to restore self-regulation processes in the absence of rest. The findings are also inconsistent with the idea of “conservation” of self-regulation resources (Muraven et al., 2006), given that recovery was observed by block 2 in experiment 1 and block 3 in Experiment 2. If participants with poor accuracy were conserving self-regulation resources, then we would expect recovery only on block 3 for both experiments. The results of the present study align with Converse and Deshon (2009), who showed self-regulation depletion with two consecutive tasks, but improvements when three consecutive self-regulation tasks were employed, suggesting that cognitive self-regulation processes can adapt given sufficient time and practice. Each block of the SST lasted approximately 5 min, which may be enough time for learning and practice to lead to the automation of processes, reducing reliance on self-regulation mechanisms. When the relative difference in reliance on self-regulation processes is amplified between the depleting and comparative control task (i.e., in our case a discrimination vs. a detection task), the time needed for recovery may be prolonged. Alternatively, the observed fluctuations in self-regulation across and within tasks may reflect changes in arousal levels with task difficulty as predicted by the Yerkes-Dodson law (Yerkes and Dodson, 1908; Lupien et al., 2007). Indeed previously arithmetic tasks have been shown to be effective at altering arousal levels (Peters et al., 1998; Chatkoff et al., 2010); however, recovery from both acute and chronic cognitive and physical stressors is very rapid (Chatkoff et al., 2010; Sander et al., 2010), and unlikely to transfer from the arithmetic to the SST. The availability of self-regulation resources within the cognitive domain appears to evolve rapidly with time, and further research is needed to understand the behavioral and neural mechanisms driving these dynamic changes in self-regulation resources.

A surprising finding of the present study is that self-regulation depletion generalized to effect the go-signal RT of the SST. The increase in go-signal response times was coupled with an increase in stopping delays following the arithmetic task, which may reflect strategic changes in the way the SST is performed following the arithmetic task. However, these changes did not completely counteract SSRT depletion in Experiment 2, suggesting that both SSRT and go-signal RTs are adversely affected, with the effects being more pronounced when a simple detection task is used as a comparative control. Indeed the simplest of psychophysical tasks tax multiple capacities involved in self-regulation and, in turn, have the potential to tax self-regulation resources. Asking participants to ignore equations and focus on the sign of the operation, although cognitively less engaging, still places a considerable demand on sustained attention, reducing the likelihood of seeing a difference in stop-signal task measures following the arithmetic task. Further reducing self-regulatory demands by using a simple detection task we observed an increase in go-signal RTs for both groups, which are also partly dependent on one’s ability to regulate their own behavior. This may reflect a generalization of depletion to other cognitive processes beyond self-regulation. Indeed, there is converging evidence from human electrophysiological and functional imaging studies suggesting that the go- and stop-signal in SSTs engage different frontal-parietal and frontal-striatal neural networks (e.g., Aron and Poldrack, 2006; Dimoska et al., 2006; Schmajuk et al., 2006; Alegre et al., 2008; Dimoska and Johnstone, 2008; Huster et al., 2010, 2011). Further research is needed to understand the relationship between self-regulation depletion and cognitive demands and their neural substrates.

Typically, successful self-regulation across a range of domains has been related to top-down control from prefrontal regions with the target of control being posterior cortical and subcortical regions (reviewed in Aron, 2007; Cohen and Lieberman, 2010; Heatherton and Wagner, 2011). Cognitive control tasks, such as switching tasks, are associated with neural networks of activation generally involving medial and lateral regions of the prefrontal cortex including the left dorso-lateral prefrontal cortex (DLPFC), the parietal lobe, supplementary motor areas (pre-SMA), and subcortical regions (e.g., Kimberg et al., 2000; MacDonald et al., 2000; Sohn et al., 2000; Brass and von Cramon, 2002; Rushworth et al., 2002; Monsell, 2003; Savine and Braver, 2010). Similarly, response inhibition on the SST has been associated with activity of the right inferior frontal gyrus (IFG), DLPFC, intraparietal sulcus (IPS), the pre-SMA, the globus pallidus, and the right subthalamic nucleus (STN) (e.g., Aron and Poldrack, 2006; Li et al., 2006; Chikazoe et al., 2009; Duann et al., 2009). Although switch tasks and SSTs are likely to engage different frontal-parietal and fontal-striatal networks, they do share common neural links, for example, with the prefrontal cortex. Lesions to prefrontal cortical regions have been shown to result in performance deficits on both switching tasks and the SST (Rogers et al., 1998; Keele and Rafal, 2000; Aron et al., 2004a,b; Robbins, 2007). Prefrontal brain regions have previously been shown to be susceptible to resource depletion. For example, racial bias believed to deplete executive attention resources, can predict right DLPFC activity, and changes in right DLPFC activity in white individuals after being exposed to black individuals have been shown to predict performance on a preceding unrelated color Stroop task (Richeson et al., 2003). Thus, one neural basis for the cross influence between the arithmetic switching task and the SST task observed in the present study may be the prefrontal cortex, though further research is required to validate this hypothesis.

The current findings suggest that the strength model is limited in its ability to explain the dynamic nature of self-regulation, and its ability to up-regulate without rest. These findings partly explain why people are able to withstand situations requiring extended periods of self-regulation demand. A dip in self-regulation resources may activate compensatory processes to meet environmental or situational demands. However, these effects may be dependent on the level of cognitive demand; there may be situations where self-regulation may not be able to self-recover without rest or glucose intake, which need to be investigated further. Given that self-regulation is a process that extends across multiple domains ranging from cognition cognitive control, including inhibition of behaviors and impulsivity, to social and personality traits, it is likely that self-regulation may be able to up-regulate and compensate for dips in self-regulation across other domains too.

The availability of self-regulation resources within the cognitive domain is dynamic and susceptible to various independent influences. Contrary to the claims of the majority of researchers on the topic, rest (or glucose) are not required to restore self-regulation resources. Indeed in some individuals self-regulation can even improve after engaging in a cognitively demanding task. Further research is needed to understand the dynamics self-regulatory processes as such research may help identify situations in which some people are more likely to experience lapses in their ability to self-regulate and engage in undesired or harmful behavior.

## Conflict of Interest Statement

The authors declare that the research was conducted in the absence of any commercial or financial relationships that could be construed as a potential conflict of interest.

## References

[B1] Alderson R. M. Rapport M. D. Kofler M. J. (2007). Attention-deficit/hyperactivity disorder and behavioral inhibition: a meta-analytic review of the stop-signal paradigm. J. Abnorm. Child. Psychol. 35, 745–758 10.1007/s10802-007-9131-6 17668315

[B2] Alegre M. Alvarez-Gerriko I. Valencia M. Iriarte J. Artieda J. (2008). Oscillatory changes related to the forced termination of a movement. Clin. Neurophysiol. 119, 290–300 10.1016/S1388-2457(08)60061-9 18083620

[B3] Aron A. R. (2007). The neural basis of inhibition in cognitive control. Neuroscientist. 13, 214–228 10.1177/1073858407299288 17519365

[B4] Aron A. R. Monsell S. Sahakian B. J. Robbins T. W. (2004a). A componential analysis of task-switching deficits associated with lesions of left and right frontal cortex. Brain 127, 1561–1573 10.1093/brain/awh169 15090477

[B5] Aron A. R. Robbins T. W. Poldrack R. A. (2004b). Inhibition and the right inferior frontal cortex. Trends Cogn. Sci. (Regul. Ed.) 8, 170–177 10.1016/j.tics.2004.02.010 15050513

[B6] Aron A. R. Poldrack R. A. (2006). Cortical and subcortical contributions to stop signal response inhibition: role of the subthalamic nucleus. J. Neurosci. 26, 2424–2433 10.1523/JNEUROSCI.4682-05.2006 16510720 PMC6793670

[B7] Band G. P. Van Der Molen M. W. Logan G. D. (2003). Horse-race model simulations of the stop-signal procedure. Acta Psychol. (Amst) 112, 105–142 10.1016/S0001-6918(02)00079-3 12521663

[B8] Baumeister R. F. Bratslavsky E. Muraven M. Tice D. M. (1998). Ego depletion: is the active self a limited resource? J. Pers. Soc. Psychol. 74, 1252–1265 10.1037/0022-3514.74.5.1252 9599441

[B9] Baumeister R. F. Gailliot M. Dewall C. N. Oaten M. (2006). Self-regulation and personality: how interventions increase regulatory success, and how depletion moderates the effects of traits on behavior. J. Pers. 74, 1773–1801 10.1111/j.1467-6494.2006.00428.x 17083666

[B10] Baumeister R. F. Tierney J. (2011). Willpower: Rediscovering the Greatest Human Strength. New York: The Penguin Press

[B11] Baumeister R. F. Vohs K. D. (2007). Self-regulation, ego depletion, and motivation. Soc. Personal. Psychol. Compass 1, 1–14 10.1111/j.1751-9004.2007.00008.x

[B12] Berryhill M. E. Hughes H. C. (2009). On the minimization of task switch costs following long-term training. Atten. Percept. Psychophys. 71, 503–514 10.3758/APP.71.3.503 19304641

[B13] Brass M. von Cramon D. Y. (2002). The role of the frontal cortex in task preparation. Cerebr. Cortex 12, 908–914 10.1093/cercor/12.9.908 12183390

[B14] Chatkoff D. K. Maier K. J. Klein C. (2010). Nonlinear associations between chronic stress and cardiovascular reactivity and recovery. Int. J. Psychophysiol. 77, 150–156 10.1016/j.ijpsycho.2010.05.008 20561895

[B15] Chikazoe J. Jimura K. Hirose S. Yamashita K. Miyashita Y. Konishi S. (2009). Preparation to inhibit a response complements response inhibition during performance of a stop-signal task. J. Neurosci. 29, 15870–15877 10.1523/JNEUROSCI.3645-09.2009 20016103 PMC6666181

[B16] Cohen J. D. Lieberman M. D. (2010). “The common neural basis of exerting self-control in multiple domains,” in Self Control in Society, Mind and Brain, eds Hassin R. Ochsner K. Trope Y. (Oxford: Oxford University Press), 141–160 10.1093/acprof:oso/9780195391381.003.0008

[B17] Converse P. D. Deshon R. P. (2009). A tale of two tasks: reversing the self-regulatory resource depletion effect. J. Appl. Psychol. 94, 1318–1324 10.1037/a0014604 19702373

[B18] Dimoska A. Johnstone S. J. (2008). Effects of varying stop-signal probability on ERPs in the stop-signal task: do they reflect variations in inhibitory processing or simply novelty effects? Biol. Psychol. 77, 324–336 10.1016/j.biopsycho.2007.11.005 18096294

[B19] Dimoska A. Johnstone S. J. Barry R. J. (2006). The auditory-evoked N2 and P3 components in the stop-signal task: indices of inhibition, response-conflict or error-detection? Brain Cogn. 62, 98–112 10.1016/j.bandc.2006.03.011 16814442

[B20] Duann J. R. Ide J. S. Luo X. Li C. S. (2009). Functional connectivity delineates distinct roles of the inferior frontal cortex and presupplementary motor area in stop signal inhibition. J. Neurosci. 29, 10171–10179 10.1523/JNEUROSCI.1300-09.2009 19675251 PMC2769086

[B21] Gailliot M. T. Baumeister R. F. (2007). The physiology of willpower: linking blood glucose to self-control. Pers. Soc. Psychol. Rev. 11, 303–327 10.1177/1088868307303030 18453466

[B22] Gailliot M. T. Baumeister R. F. Dewall C. N. Maner J. K. Plant E. A. Tice D. M. (2007). Self-control relies on glucose as a limited energy source: willpower is more than a metaphor. J. Pers. Soc. Psychol. 92, 325–336 10.1037/0022-3514.92.2.325 17279852

[B23] Glenville M. Broughton R. (1978). Reliability of the Stanford Sleepiness Scale compared to short duration performance tests and the Wilkinson Auditory Vigilance Task. Adv. Biosci. 21, 235–244 755721

[B24] Goldberg L. S. Grandey A. A. (2007). Display rules versus display autonomy: emotion regulation, emotional exhaustion, and task performance in a call center simulation. J. Occup. Health Psychol. 12, 301–318 10.1037/1076-8998.12.3.301 17638495

[B25] Hagger M. S. Wood C. Stiff C. Chatzisarantis N. L. (2010). Ego depletion and the strength model of self-control: a meta-analysis. Psychol. Bull. 136, 495–525 10.1037/a0019486 20565167

[B26] Heatherton T. F. Wagner D. D. (2011). Cognitive neuroscience of self-regulation failure. Trends Cogn. Sci. (Regul. Ed.) 15, 132–139 10.1016/j.tics.2010.12.005 21273114 PMC3062191

[B27] Hofmann W. Vohs K. D. Baumeister R. F. (2012). What people desire, feel conflicted about, and try to resist in everyday life. Psychol. Sci. 23, 582–588 10.1177/0956797612437426 22547657

[B28] Huster R. J. Eichele T. Enriquez-Geppert S. Wollbrink A. Kugel H. Konrad C. (2011). Multimodal imaging of functional networks and event-related potentials in performance monitoring. Neuroimage 56, 1588–1597 10.1016/j.neuroimage.2011.03.039 21421060

[B29] Huster R. J. Westerhausen R. Pantev C. Konrad C. (2010). The role of the cingulate cortex as neural generator of the N200 and P300 in a tactile response inhibition task. Hum. Brain Mapp. 31, 1260–1271 20063362 10.1002/hbm.20933 PMC6871040

[B30] Job V. Dweck C. S. Walton G. M. (2010). Ego depletion – is it all in your head? implicit theories about willpower affect self-regulation. Psychol. Sci. 21, 1686–1693 10.1177/0956797610384745 20876879

[B31] Jonker L. Elferink-Gemser M. T. Toering T. T. Lyons J. Visscher C. (2010). Academic performance and self-regulatory skills in elite youth soccer players. J. Sports Sci. 28, 1605–1614 10.1080/02640411003797157 21104520

[B32] Keele S. W. Rafal R. (2000). “Deficits of task-set in patients with left prefrontal cortex lesions,” in Control of Cognitive Processes: Attention and Perfromance XVIII, eds. Monsell S. Driver J. S. (Cambridge, MA: MIT Press), 627–652

[B33] Kiesel A. Steinhauser M. Wendt M. Falkenstein M. Jost K. Philipp A. M. (2010). Control and interference in task switching – a review. Psychol. Bull. 136, 849–874 10.1037/a0019842 20804238

[B34] Kimberg D. Y. Aguirre G. K. D’Esposito M. (2000). Modulation of task-related neural activity in task-switching: an fMRI study. Brain Res. Cogn. Brain Res. 10, 189–196 10.1016/S0926-6410(00)00016-1 10978708

[B35] Levy N. (2011). “Addiction, responsibility and ego-depletion,” in Addiction and Responsibility, eds Poland J. Graham G. (Cambridge, MA: MIT Press), 89–111

[B36] Li C. S. Huang C. Constable R. T. Sinha R. (2006). Imaging response inhibition in a stop-signal task: neural correlates independent of signal monitoring and post-response processing. J. Neurosci. 26, 186–192 10.1523/JNEUROSCI.3467-06.2006 16399686 PMC6674298

[B37] Logan G. D. Schachar R. J. Tannock R. (1997). Impulsivity and inhibitory control. Psychol. Sci. 8, 60–64 10.1111/j.1467-9280.1997.tb00545.x

[B38] Lupien S. J. Maheu F. Tu M. Fiocco A. Schramek T. E. (2007). The effects of stress and stress hormones on human cognition: implications for the field of brain and cognition. Brain Cogn. 65, 209–237 10.1016/j.bandc.2007.02.007 17466428

[B39] MacDonald A. W. 3rd, Cohen, J. D. Stenger V. A. Carter C. S. (2000). Dissociating the role of the dorsolateral prefrontal and anterior cingulate cortex in cognitive control. Science 288, 1835–1838 10.1126/science.288.5472.1835 10846167

[B40] Miller H. C. Pattison K. F. Dewall C. N. Rayburn-Reeves R. Zentall T. R. (2010). Self-control without a “self”?: common self-control processes in humans and dogs. Psychol. Sci. 21, 534–538 10.1177/0956797609357733 20424096

[B41] Mischel W. Shoda Y. Rodriguez M. I. (1989). Delay of gratification in children. Science 244, 933–938 10.1126/science.2658056 2658056

[B42] Monsell S. (2003). Task switching. Trends Cogn. Sci. (Regul. Ed.) 7, 134–140 10.1016/S1364-6613(03)00028-7 12639695

[B43] Muraven M. Shmueli D. Burkley E. (2006). Conserving self-control strength. J. Pers. Soc. Psychol. 91, 524–537 10.1037/0022-3514.91.3.524 16938035

[B44] Muraven M. Slessareva E. (2003). Mechanisms of self-control failure: motivation and limited resources. Pers. Soc. Psychol. Bull. 29, 894–906 10.1177/0146167203029007008 15018677

[B45] Muraven M. Tice D. M. Baumeister R. F. (1998). Self-control as limited resource: regulatory depletion patterns. J. Pers. Soc. Psychol. 74, 774–789 10.1037/0022-3514.74.3.774 9523419

[B46] Peters M. L. Godaert G. L. Ballieux R. E. Van Vliet M. Willemsen J. J. Sweep F. C. (1998). Cardiovascular and endocrine responses to experimental stress: effects of mental effort and controllability. Psychoneuroendocrinology 23, 1–17 10.1016/S0306-4530(97)00082-6 9618748

[B47] Richeson J. A. Baird A. A. Gordon H. L. Heatherton T. F. Wyland C. L. Trawalter S. (2003). An fMRI investigation of the impact of interracial contact on executive function. Nat. Neurosci. 6, 1323–1328 10.1038/nn1156 14625557

[B48] Robbins T. W. (2007). Shifting and stopping: fronto-striatal substrates, neurochemical modulation and clinical implications. Philos. Trans. R. Soc. Lond. B Biol. Sci. 362, 917–932 10.1098/rstb.2007.2097 17412678 PMC2430006

[B49] Rogers R. D. Sahakian B. J. Hodges J. R. Polkey C. E. Kennard C. Robbins T. W. (1998). Dissociating executive mechanisms of task control following frontal lobe damage and Parkinson’s disease. Brain 121(Pt 5), 815–842 10.1093/brain/121.5.815 9619187

[B50] Rushworth M. F. Hadland K. A. Paus T. Sipila P. K. (2002). Role of the human medial frontal cortex in task switching: a combined fMRI and TMS study. J. Neurophysiol. 87, 2577–2592 11976394 10.1152/jn.2002.87.5.2577

[B51] Sander M. Macefield V. G. Henderson L. A. (2010). Cortical and brain stem changes in neural activity during static handgrip and postexercise ischemia in humans. J. Appl. Physiol. 108, 1691–1700 10.1152/japplphysiol.91539.2008 20185626

[B52] Savine A. C. Braver T. S. (2010). Motivated cognitive control: reward incentives modulate preparatory neural activity during task-switching. J. Neurosci. 30, 10294–10305 10.1523/JNEUROSCI.2052-10.2010 20685974 PMC2935640

[B53] Schmajuk M. Liotti M. Busse L. Woldorff M. G. (2006). Electrophysiological activity underlying inhibitory control processes in normal adults. Neuropsychologia 44, 384–395 10.1016/j.neuropsychologia.2005.06.005 16095637

[B54] Schmeichel B. J. (2007). Attention control, memory updating, and emotion regulation temporarily reduce the capacity for executive control. J. Exp. Psychol. Gen. 136, 241–255 10.1037/0096-3445.136.2.241 17500649

[B55] Schmeichel B. J. Vohs K. D. Baumeister R. F. (2003). Intellectual performance and ego depletion: role of the self in logical reasoning and other information processing. J. Pers. Soc. Psychol. 85, 33–46 10.1037/0022-3514.85.1.33 12872883

[B56] Schneider D. W. Anderson J. R. (2010). Asymmetric switch costs as sequential difficulty effects. Q. J. Exp. Psychol. (Colchester) 63, 1873–1894 10.1080/17470211003624010 PMC2908715 20401811

[B57] Shamosh N. A. Gray J. R. (2007). The relation between fluid intelligence and self-regulation depletion. Cognit. Emot. 21, 1833–1843 10.1080/02699930701273658

[B58] Sohn M. H. Ursu S. Anderson J. R. Stenger V. A. Carter C. S. (2000). The role of prefrontal cortex and posterior parietal cortex in task switching. Proc. Natl. Acad. Sci. U.S.A. 97, 13448–13453 10.1073/pnas.200361297 11069306 PMC27244

[B59] Stewart C. C. Wright R. A. Hui S. K. Simmons A. (2009). Outcome expectancy as a moderator of mental fatigue influence on cardiovascular response. Psychophysiology 46, 1141–1149 10.1111/j.1469-8986.2009.00862.x 19572905

[B60] Tice D. M. Baumeister R. F. Shmueli D. Muraven M. (2007). Restoring self-control: positive affect helps improve self-regulation following ego depletion. J. Exp. Soc. Psychol. 43, 379–384 10.1016/j.jesp.2006.05.007

[B61] Tyler J. M. Burns K. C. (2008). After depletion: the replenishment of the self’s regulatory resources. Self Identify 7, 305–321 10.1080/15298860701799997

[B62] Vohs K. D. Baumeister R. F. Ciarocco N. J. (2005). Self-regulation and self-presentation: regulatory resource depletion impairs impression management and effortful self-presentation depletes regulatory resources. J. Pers. Soc. Psychol. 88, 632–657 10.1037/0022-3514.88.4.632 15796665

[B63] Vohs K. D. Heatherton T. F. (2000). Self-regulatory failure: a resource-depletion approach. Psychol. Sci. 11, 249–254 10.1111/1467-9280.00250 11273412

[B64] Wright R. A. Martin R. E. Bland J. L. (2003). Energy resource depletion, task difficulty, and cardiovascular response to a mental arithmetic challenge. Psychophysiology 40, 98–105 10.1111/1469-8986.40.s1.3 12751807

[B65] Wright R. A. Stewart C. C. Barnett B. R. (2008). Mental fatigue influence on effort-related cardiovascular response: extension across the regulatory (inhibitory)/non-regulatory performance dimension. Int. J. Psychophysiol. 69, 127–133 10.1016/j.ijpsycho.2008.04.002 18499290

[B66] Wylie G. Allport A. (2000). Task switching and the measurement of “switch costs.” Psychol. Res. 63, 212–233 10.1007/s004269900003 11004877

[B67] Yerkes R. M. Dodson J. D. (1908). The relation of strength of stimulus to rapidity of habit formation. J. Comparat. Neurol. Psychol. 2, 160–168

